# Fusion of encoder-decoder deep networks improves delineation of multiple nuclear phenotypes

**DOI:** 10.1186/s12859-018-2285-0

**Published:** 2018-08-07

**Authors:** Mina Khoshdeli, Garrett Winkelmaier, Bahram Parvin

**Affiliations:** Electrical and Biomedical Department, University of Nevada, Reno, 1664 N. Virginia, Reno, USA

**Keywords:** Computational histopathology, Deep learning, Nuclear segmentation

## Abstract

**Background:**

Nuclear segmentation is an important step for profiling aberrant regions of histology sections. If nuclear segmentation can be resolved, then new biomarkers of nuclear phenotypes and their organization can be predicted for the application of precision medicine. However, segmentation is a complex problem as a result of variations in nuclear geometry (e.g., size, shape), nuclear type (e.g., epithelial, fibroblast), nuclear phenotypes (e.g., vesicular, aneuploidy), and overlapping nuclei. The problem is further complicated as a result of variations in sample preparation (e.g., fixation, staining). Our hypothesis is that (i) deep learning techniques can learn complex phenotypic signatures that rise in tumor sections, and (ii) fusion of different representations (e.g., regions, boundaries) contributes to improved nuclear segmentation.

**Results:**

We have demonstrated that training of deep encoder-decoder convolutional networks overcomes complexities associated with multiple nuclear phenotypes, where we evaluate alternative architecture of deep learning for an improved performance against the simplicity of the design. In addition, improved nuclear segmentation is achieved by color decomposition and combining region- and boundary-based features through a fusion network. The trained models have been evaluated against approximately 19,000 manually annotated nuclei, and object-level Precision, Recall, F1-score and Standard Error are reported with the best F1-score being 0.91. Raw training images, annotated images, processed images, and source codes are released as a part of the Additional file 1.

**Conclusions:**

There are two intrinsic barriers in nuclear segmentation to H&E stained images, which correspond to the diversity of nuclear phenotypes and perceptual boundaries between adjacent cells. We demonstrate that (i) the encoder-decoder architecture can learn complex phenotypes that include the vesicular type; (ii) delineation of overlapping nuclei is enhanced by fusion of region- and edge-based networks; (iii) fusion of ENets produces an improved result over the fusion of UNets; and (iv) fusion of networks is better than multitask learning. We suggest that our protocol enables processing a large cohort of whole slide images for applications in precision medicine.

**Electronic supplementary material:**

The online version of this article (10.1186/s12859-018-2285-0) contains supplementary material, which is available to authorized users.

## Background

**Fig. 1:**
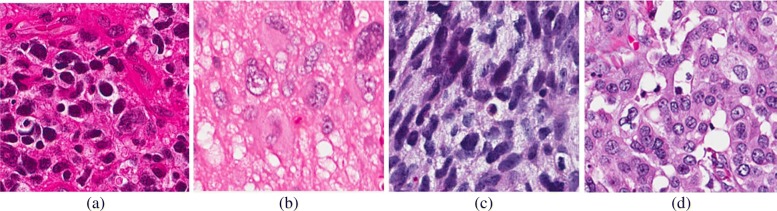
A subset of nuclear phenotypes is shown. (**a**) hyperchromatic, (**b**) pro-necrotic, (**c**) pleomorphic and invasive, and (**d**) vesicular

Nuclear morphology is an important step in identifying aberrant phenotypes in hematoxylin and eosin (H&E) stained histology sections. However, to date, the problem of nuclear segmentation, for every type of nuclear phenotype remains partially unresolved. If nuclear segmentation is preformed robustly, then malignant phenotypes can be stratified across a large cohort of histology sections. The main challenges originate from technical variations and biological heterogeneity in a large cohort. Technical variations refer to non-uniformity in sample preparations (e.g., staining), and biological heterogeneity refers to the fact that no two histology sections are alike. In most cases, technical variations are also coupled with biological heterogeneity, which complicates the construction of a stable computational model for nuclear segmentation. The diversity of the nuclear phenotypes originates from many factors. For example, (i) cancer cells tend to be larger than normal cells, and if coupled with high chromatin content, they may indicate aneuploidy; (ii) nuclei may have vesicular phenotypes; (iii) nuclei may have high pleomorphism in tumor sections; (iv) cells may be going through apoptosis or necrosis; (v) cell cytoplasm may be lost as a result of clear cell carcinoma; and (vi) cellular phenotypes may be altered as a result of macromolecules being secreted into the microenvironment. Samples of these phenotypes are shown in Fig. 1. These phenotypes suggest complexities that are associated with nuclear segmentation as one of the steps toward profiling of histology sections for diagnostics or discovery of new biomarkers. Because of the complexities associated with vesicular phenotypes, most of the previous segmentation literature has focused on nuclear phenotypes having high DNA content. However, we show that simultaneous delineation of vesicular and other phenotypes can be achieved with fusion of the deep learning models.

In recent years, convolutional neural networks (CNN)s have emerged as the most powerful technique for image classification [1, 2], and image segmentation [3–5]. CNNs can be continuously trained and improved as the number of annotated training samples increases. Furthermore, their architecture is modular, where each module can be trained for different image-based representation, and modules can be integrated to improve the outcome. Typical applications of CNNs have been used to perform image classification in the computer vision literature. CNN consists of several layers of convolution operation, where each convolutional layer is usually followed by max-pooling. The last layer is a fully connected layer, which maps a high dimensional vector to a low dimensional probability vector corresponding to distinct classes. A diversity of CNN architectures has been proposed based on the depth and size of the model for the classification ImageNet [2], VGG [1], and ResNet [6]. The segmentation task can also be performed by using a sliding window coupled with the classification for labeling each pixel in the image. However, this approach has been shown to be either noisy, less accurate, or time-consuming. To overcome these issues, alternative CNN architectures (e.g., FCN[3], UNet[5], SegNet[7], ENet[4]), based on an encoder-decoder architecture, have been proposed for region-based segmentation. The encoder architecture is the same as vanilla CNN, which consists of several convolution layers followed by max-pooling. The encoder layers perform feature extraction and region-based classification of the down-sampled image. On the other hand, the decoder layers perform up-sampling after each convolutional layer, to compensate the down-sampling effects of the encoder, and, to generate an output with the same size as the input. Some of these models are symmetric (e.g., the encoder and decoder have the same depth) and some are asymmetric. In the latter case, the decoder has the advantage of the smaller number of convolutional layers for reducing the computational load.

There are two comprehensive review papers on nuclear segmentation techniques [8, 9]; therefore, we limit ourselves to a summary here, which span from simple thresholding to the application of convolutional neural networks.

The most popular nuclear segmentation approaches include thresholding following morphological operations [10–12], watershed [13], deformable models [14], and graph-based models [15, 16] or a combination of these methods. In [11], images are binarized, morphological operators are applied, and nuclear features have been computed to profile the tumor morphology. In [13], the watershed segmentation has been applied to the magnitude of the gradient image, where the initial seeds have been generated by morphological operations. This technique is very dependent on the initial seeds, and over-segmentation may occur due to non-uniform nuclear regions. In [14], an efficient active contour model was proposed; however, this technique would not work well for nuclei having a vesicular phenotype. Similar methods have also been proposed with multi-step graph cut formulation [16], but the key assumption remains about nuclei with high chromatin content. In [15], Gaussian Mixture Model (GMM) of nuclear phenotypes were constructed by annotating nuclear regions. The GMM representation was based on the Laplacian of Gaussian (LoG) response and the RGB values in the color space. Next, a multi-reference graph cut method was developed to binarize the image. Subsequently, each clump of overlapping nuclei was partitioned using geometric reasoning. Because this technique is model-based, intrinsic variations of DNA content are captured in GMM. A similar method was proposed in [17]; however, the threshold parameter was learned by training an support vector machine (SVM) model. Graph cut has the additional advantage over SVM since it incorporates spatial consistency. In summary, except for the last two methods, most classical techniques are procedural and model-free with a large number of free parameters.

Applications of CNNs to medical images have been recently reviewed [18, 19] and include (i) nuclear detection and segmentation in pathology images, (ii) tumor delineation from the MRI data, and (iii) extraction of anatomical structures from the tomography data. Because of our focus on pathology, several relevant techniques on nuclear detection and segmentation, and gland segmentation are summarized below. (a) With respect to nuclear detection, three strategies are reviewed here. In [20], a spatially constrained CNN model has been trained for nuclear detection. The model has been spatially constrained by assigning a higher probability to the pixels that are closer to the centroids of nuclei. A similar approach has been proposed in [21], where a CNN model is trained to generate the positions of the nuclei and their corresponding confidence in a given patch. In [22], a CNN model has been trained with the feature-based representation of the original image based on the Laplacian of Gaussian (LoG) filter response. The advantage of the LoG filter is that it accentuates the blob-shape of nuclei and provides an approximate location of each nucleus. This approach has been applied to detect various types of the nuclear phenotype. (b) With respect to nuclear segmentation, CNN models have been trained for region-based segmentation, semantic-level feature extraction, and nuclear segmentation. In [23], an active contour model has been utilized for nuclear segmentation in H&E stained breast histology sections, and a CNN model has been trained to extract semantic-level features and to make an initial classification of the image into the low, intermediate, or high-grade tumor. Subsequently, the final classification is refined by integrating semantic-level (e.g., the ratio of nuclei belonging to different grades), colony organization level (e.g., the relationship of nuclei within and across colonies), and pixel-level (e.g., texture) features to train an SVM. However, the active contour model assumes that nuclei are well isolated and have high chromatin, which is not necessarily the case. In fact, for breast cancer, nuclear atypia is one of the visual representation for grading. In [24], a CNN-based model has been proposed for nuclei segmentation from H&E stained sections, where the CNN is trained to classify each pixel to be nuclei or non-nuclei. In [25], a multiscale convolutional network has been proposed for the segmentation of the cervical cytoplasm and nuclei. The multiscale CNN incorporates a pyramid image representation for initial pixel-based classification. Next graphcut is applied since CNN does not enforce spatial continuity. Finally, segmentation results are refined by morphological operators such as a marker-based watershed. In [26], nuclear segmentation has been performed by converting the RGB image into gray scale, denoising the image, and applying the CNN to separate background and foreground. Finally, nuclear segmentation is refined by morphological operators. A similar approach has been proposed in [27], a CNN based model has been trained to provide the initial probability map for nuclear segmentation. Then, a deformable shape model has been applied to separate overlapping nuclei. In [28], a vanilla convolutional neural network has been proposed, which consists of 3 convolutional, 3 pooling, and 2 fully connected layers. This model is not an end-to-end segmentation network, since there is a single output label for an input image. Therefore, a sliding window technique is required to compute the segmentation output, which is time consuming. In [29], a fully convolutional neural network has been proposed for nuclei segmentation. The model architecture is similar to the UNet [5] model, which is a symmetric encoder-decoder network. The model has been trained to segment the nuclear regions and the boundary of nuclei simultaneously. In [30], we proposed an earlier version of the fusion framework for segmentation of nuclei, which integrates the region and boundary information using the ENet models. (c) With respect to gland segmentation, two papers are reviewed here. In [31], a multichannel convolutional network has been trained for gland segmentation. Separate convolutional modules have been used for edge detection and region segmentation and a shallow CNN fused the edge and region information. In [32], the FCN [3] model has been trained for gland segmentation. The authors applied the concept of multi-task learning to use the same weights for the region and boundary segmentation.

## Method

**Fig. 2:**
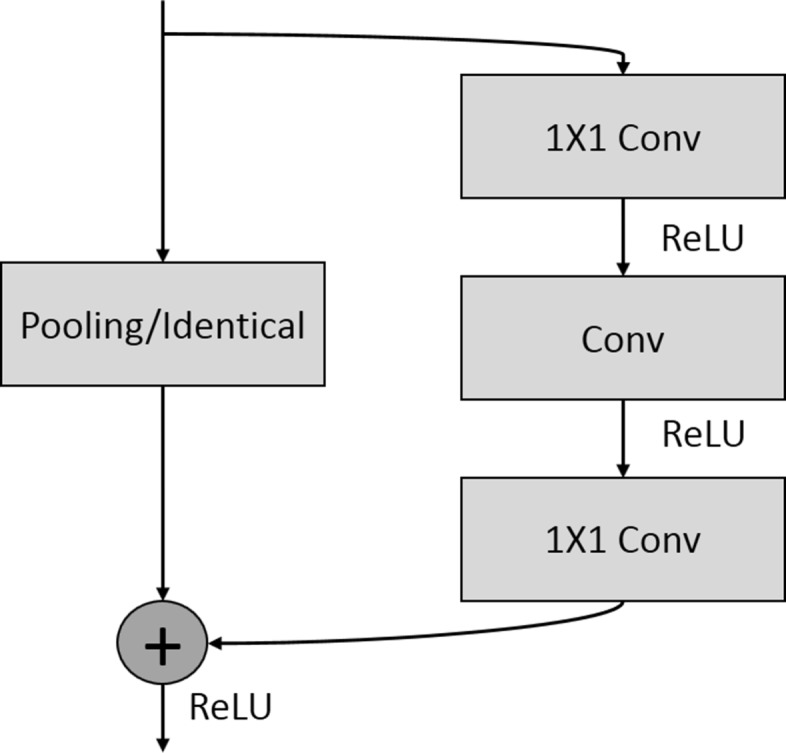
The building block of the encoder-decoder network utilizes either 5-by-5, 3-by-3, or 1-by-1 convolution. The pooling performs down-sampling in the encoder module and up-sampling in the decoder module

**Fig. 3:**
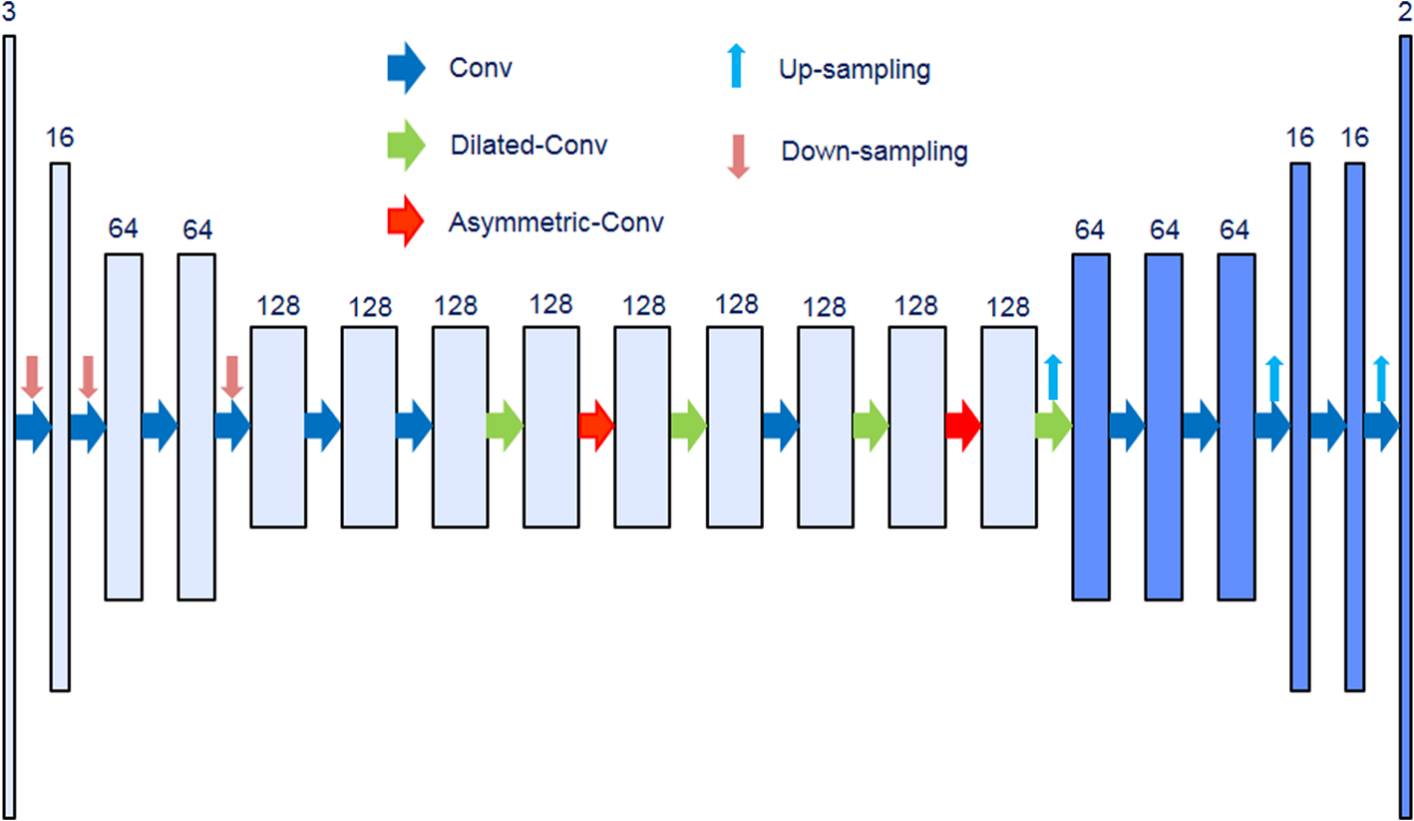
The complete architecture of the ENet model is shown. The model includes both encoder (light blue) and decoder (dark blue) parts. The upward and downward arrows indicate up-sampling and down-sampling operations. Right hand arrows show different types of convolution including normal, dilated, and asymmetric

The overall strategy is first to identify an encoder-decoder model that can best delineate diverse nuclear phenotypes, and then extend this model to improve delineation of overlapping nuclei. For simplicity, the strategy will use the same building block at each step. The building block is a composition of several convolutional, pooling, and identity layers, as shown in Fig. 2. All convolution operations are either 3-by-3, 5-by-5, or 1-by-1, where the 1-by-1 convolutions are used for reducing the dimension of the feature maps. The 5-by-5 convolutions are replaced with 5-by-1 and 1-by-5 convolutions to reduce the computational loads. In the encoder, the pooling performs down-sampling, while in the decoder the pooling performs up-sampling. We start with a shallow model that has only 3 blocks for the encoder and 3 blocks for the decoder, and increase the number of blocks to 10. The number of feature maps is 32 for the initial block, 64 for the middle block, and 128 for the last block. Finally, we will compare proposed models with the ENet architecture [4], which is shown in Fig. 3.

**Fig. 4:**
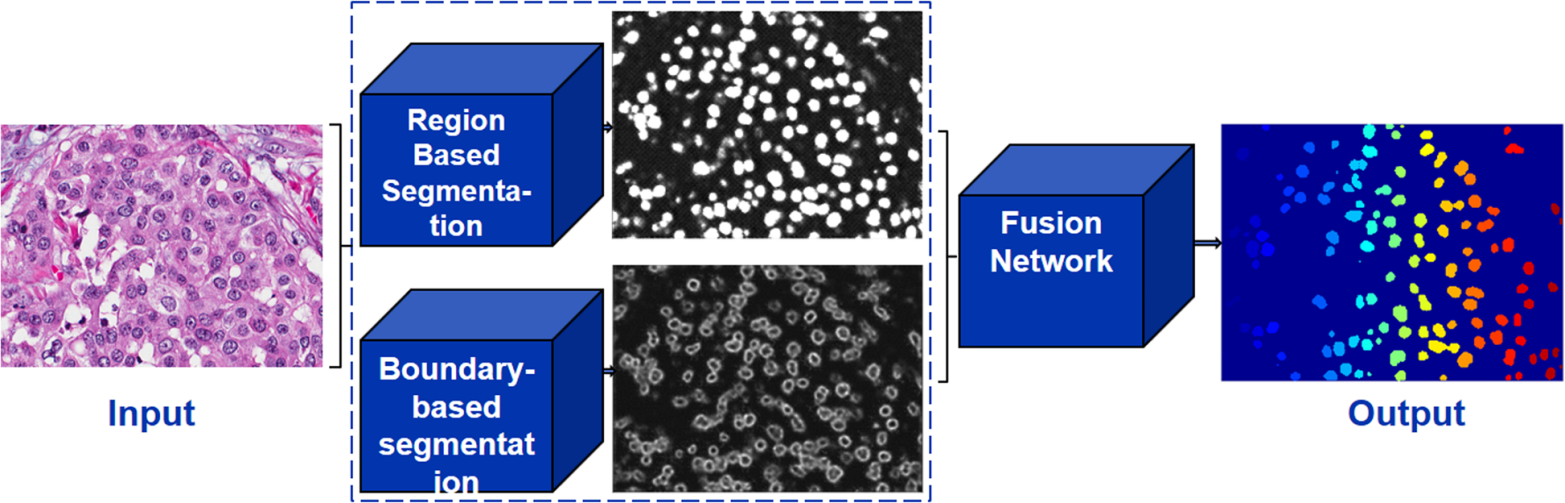
Framework for nuclear segmentation consists of three encoder-decoder networks. Two are used for region-based and edge-based segmentation. The outputs of these two networks are then fused through a third network

Having identified nuclear phenotypes with the best performing model, we will then extend this model with boundary-based representation to delineate overlapping nuclei. The main goal is to use boundary information to capture the perceptual boundaries of overlapping nuclei. Ultimately, we hypothesize that the fusion of the region- and boundary-based networks, shown in Fig. 4, improves the overall segmentation results.

### Experimental set-up

We have sampled from the whole slide images (WSIs) of the publicly available brain tumor data from The Cancer Genome Atlas (TCGA) and a Scandinavian breast cancer cohort. WSIs have been anonymized with respect to the patient identity, and a total of 32 WSIs have been selected for this study. Each WSI (i) belongs to a unique patient, (ii) is selected to reflect the diversity of the phenotypic signatures, and (iii) is scanned with a resolution of 0.5 microns per pixel. From each WSI, an image (e.g., a pinhole) is cropped and used for annotation. These images are a superset of a previously annotated cohort [15]. Manual annotations of sampled images have produced approximately 19,000 nuclei with diverse phenotypic signatures that are released as Additional file 1. The annotated data are partitioned equally (i.e. 50% -50%) into training and testing in such a way that (a) there is no overlap, and (b) the diversity of phenotypic signatures are represented in both training and testing.

### Evaluation

The proposed method is evaluated in terms of object-level *Precision*, *Recall*, and *F*1_−_*score* defined bellow.

1 $$\begin{array}{@{}rcl@{}} Precision = \frac{\#True Positive}{\#True Positive+\#False Positive} \end{array} $$

2 $$ Recall = \frac{\#True Positive}{\#True Positive+\#False Negative}  $$

3 $$ F1_{-}score = \frac{2*Precision*Recall}{Precision+Recall}  $$

Where a nucleus has been considered a true positive, when the intersection over union (IoU) of the ground truth and segmented nucleus is more than 50%. *IoU* of two sets of pixels *A* and *B* is defined as below.

4 $$ IoU(A,B) = \frac{A\cap B}{A\cup B}  $$

We have also evaluated segmentation accuracy by its standard error, which is computed by enumerating over different permutations of training and testing data.

5 $$\begin{array}{@{}rcl@{}} Standard Error = \frac{\sigma}{\sqrt{n}} \end{array} $$

Where *σ* is the standard deviation of the difference between the output and ground truth and *n* is the total number of pixels.

### Pre-processing and Training of Networks

The training process is preceded by color decomposition (CD), which decomposes the RGB signal into two channels of information corresponding to the DNA and protein contents, where the former channel is used for subsequent processing. CD is based on a recently published method that has been shown to provide superior results [22].

Trainings of the region- and boundary-based representations are independent and is followed by training of the fusion network. The region-based training relies on the annotated mask. However, training of the boundary-based representation is based on computed boundaries from the annotated masks. The Adam optimization algorithm [33] has been used for training, and the batch size (e.g., number of samples being processed at the same time) is set at four due to the limitations of the GPU memory. The learning rate and L2 weight decay were set at 5e-4 and 2e-4, respectively. The dropout method is used to avoid overfitting. The training of each model took approximately three and a half hours using one GPU card. The testing time for each 1k-by-1k image is less than 60 ms. The fusion architecture is the same as the region- and boundary-based models. Moreover, we have used the same set of parameters for training the fusion network (e.g., learning rate, the ratio of training and testing datasets).

### Post-processing

The fusion model improves the segmentation results in a number of ways that includes separation of touching nuclei; however, not all of the overlapping nuclei are delineated. Therefore, a post-processing step of the marker-based watershed method is added because of its computational simplicity and open source availability.

## Results and discussion

Our approach is to evaluate alternative architectures, starting from shallow to deep networks, and then add different complementary representations to improve performance. The rationale being that the encoder-decoder architectures can capture the diverse nuclear phenotypes, which are extremely difficult with the traditional model-based approaches. Having segmented complex phenotypic signatures, we will then use boundary information to help in delineating touching nuclei. The design, for both region- and boundary-based networks, is based on symmetric and asymmetric encoder-decoder architectures by increasing the number of layers and testing the performance incrementally. The goal is to inquire whether the nuclear segmentation problem can be improved with (i) a shallower network or deeper networks, (ii) the fusion of networks as shown in Fig. 4, or (iii) multitask learning. All of our experiments are initiated by the color decomposition method of [22] that was shown to perform better than the prior state of the art. One of the main benefits of color decomposition (CD) is that the training set can be reduced as a result of the dimensionality reduction of the raw data. To train and validate this architecture, we have annotated 19,000 nuclei from 32 different patients. Annotated nuclei originate from 32 images, each image is cropped from a pinhole of a unique whole slide image (WSI), and nuclei from the same image are never used for both training and testing.

We have performed a number of simulations, for alternative architectures, and the results are shown in Tables 1, 2, and 3. The overall approach is to evaluate a model for region-based segmentation, with a varying number of layers independently, and then select the best model for the fusion and comparison with the original model, i.e., the original asymmetric ENet model. The contour-based model does not contribute to the segmentation of different phenotypes but helps with separating adjacent nuclei. Table 1 shows that the ENet, with the original number of layers, has the best performance. Table 2 indicates that fused ENet has the best performance, on the same dataset, when compared to the previous approach[15]. Table 3 compares the performance of the fused ENet with UNet, fused UNet, and multitask learning. Our observations are summarized below.

**Table 1:** Quality of region-based nuclear segmentation remains mostly stationary as a function of increased network complexity

Input size	Recall	Precision	F1-Score	Standard Error
3-module encoder, 3-module decoder	0.80	0.89	*0.84*	1.4524e-06
4-module encoder, 4-module decoder	0.73	*0.91*	0.80	1.5081e-06
5-module encoder, 5-module decoder	0.74	0.90	0.80	1.5126e-06
6-module encoder, 6-module decoder	0.75	0.90	0.81	1.5325e-06
7-module encoder, 7-module decoder	0.76	0.90	0.81	1.5172e-06
8-module encoder, 8-module decoder	0.77	0.90	0.82	1.5004e-06
9-module encoder, 9-module decoder	0.78	0.90	0.82	1.5131e-06
10-module encoder, 10-module decoder	0.77	0.87	0.80	1.5231e-06
ENet	*0.83*	0.88	*0.84*	*1.1412e-06*

The italic items illustrate the best results

**Table 2:** Comparison of nuclear segmentation between different fusion models and a previously published method

Input size	Recall	Precision	F1-Score	Standard Error
Fused-ENet	*0.94*	*0.88*	*0.91*	*1.1124e-06*
Fused 3-module encoder, 3-module decoder	0.79	0.88	0.82	1.6170e-06
Multi-reference Graphcut (MRG)	0.75	0.85	0.79	unknown

MRG was shown to out-perform other methods that include random forest

The italic items illustrate the best results

**Table 3:** Comparison of nuclear segmentation between the fused ENet with UNet, fused UNet, and multitask learning

Input size	Recall	Precision	F1-Score	Standard Error
UNet	0.86	0.81	0.83	1.2786e-06
Fused-UNet	0.89	0.86	0.87	1.1921e-06
Multi-task UNet	0.87	0.77	0.81	1.3117e-06
ENet	0.83	0.88	0.84	1.1412e-06
Fused-ENet	*0.94*	*0.88*	*0.91*	*1.1124e-06*
Multi-task ENet	0.89	0.83	0.86	1.2690e-06

The italic items illustrate the best results

**Fig. 5:**
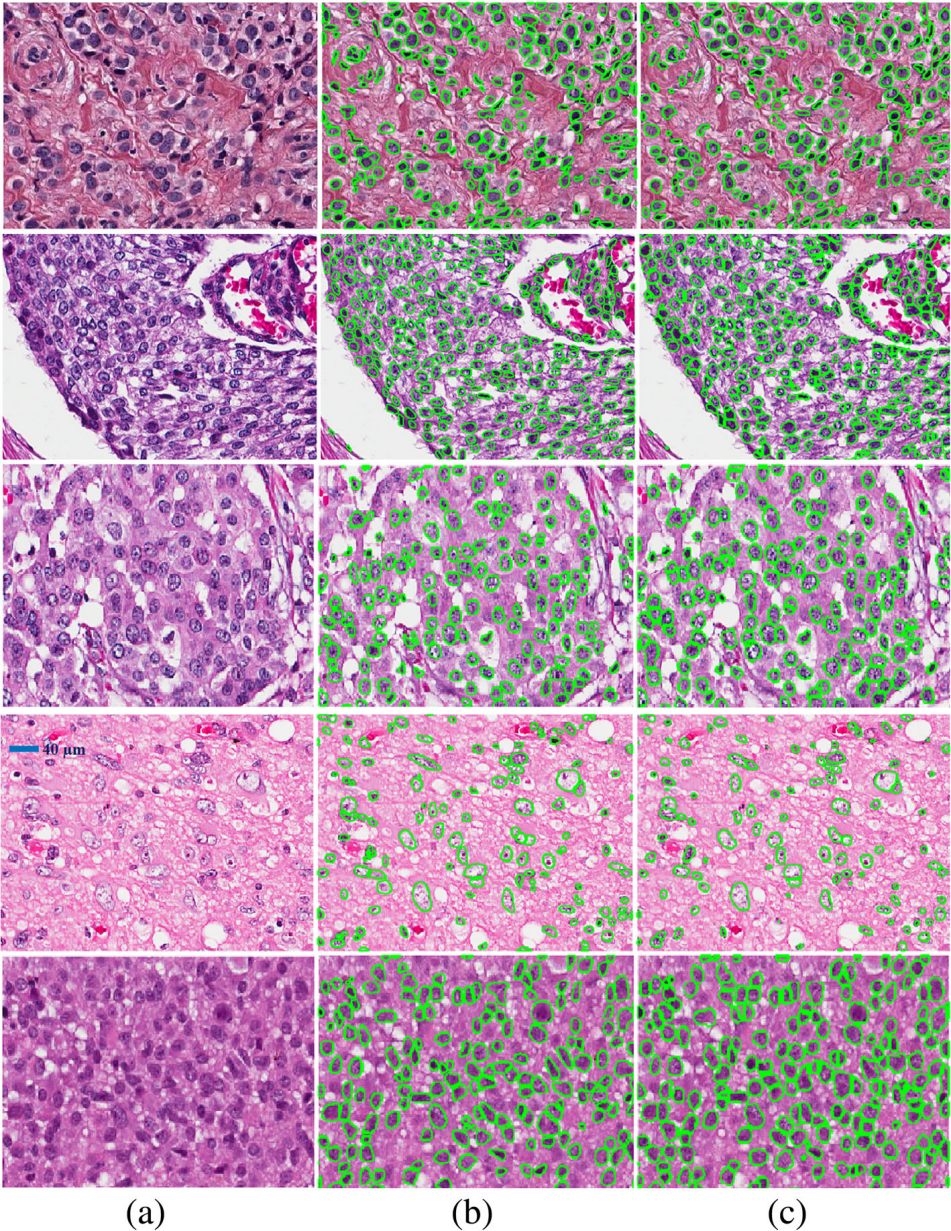
Qualitative performance of nuclear segmentation is shown for three different phenotypes. Columns (**a**), (**b**), and (**c**) illustrate the original image, ground truth, and segmentation results of the proposed model, respectively. Rows 1 and 2 correspond to sections from breast cancer tumor, and row 3 corresponds to a section from brain tumor. These images show large variations in color and phenotypic signatures. Row 1 shows a section with distinct stains of pink and blue, rows 2 shows a section with predominantly deep blue stain, and row 3 shows nuclei with vesicular phenotypes

*Improved delineation for a diversity of phenotypic signatures is observed:* One of the main challenges in nuclear segmentation has been the complexities that are associated with alternative phenotypes, as shown in Fig. 5. The problem is further complicated as a result of technical variations such as fixation and staining (e.g., the batch effects). While it is possible to engineer and handcraft a model, based on appearance and morphometry of hyperchromatic nuclei, such an approach does not extend to other phenotypes, i.e., vesicular ones. At some level, the encoder-decoder architectures are specialized filters that learn particular spatial distributions by example. The net result is that the development costs are shifted more toward curating a training dataset. More interestingly, our simulations show that a three-layer encoder-decoder architecture has the same performance as the deeper architecture of the ENet. Hence, the intrinsic diversity of nuclear phenotype, based on their spatial signatures, can be captured with a few layers.

**Fig. 6:**
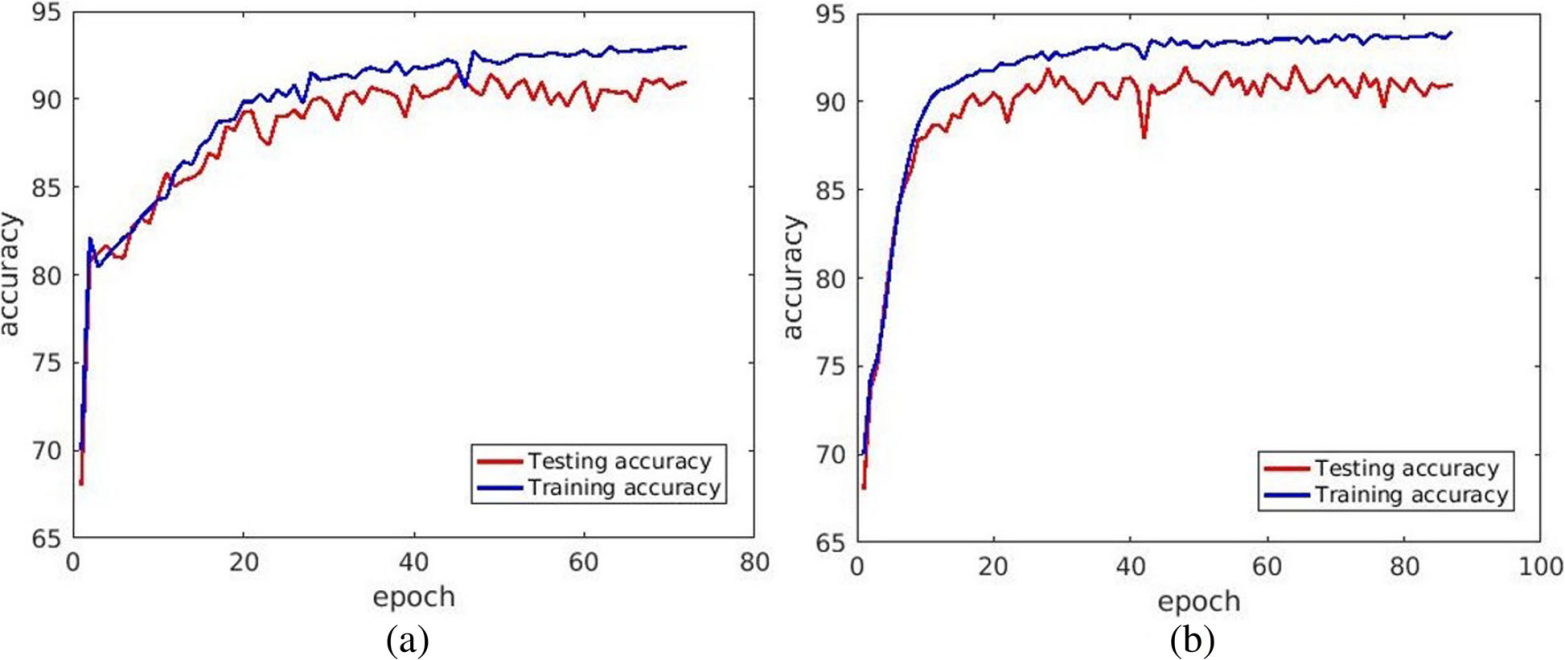
Color decomposition (CD) accelerates the training time for model construction as a function of the number of epochs required to achieve the same accuracy. Training accuracy as a function number of epochs when the input is represented as (**a**) an RGB image, and (**b**) gray scale following CD

*Color decomposition accelerates training for model construction:* Color decomposition in H&E stained images is difficult for a number of reasons that were stated earlier. Our recent approach [22] is the best performing color decomposition thus far. CD offers three advantages. First, it can accelerate the training time for model construction. Second, it reduces the required number of training samples because of the lower dimensionality of data. Third, it places the attention on pertinent nuclei morphometry and texture because the DNA content are the primary relevant information. Figure 6 shows the accuracy as a function of number of epochs for model construction using RGB and gray scale image following CD. It is clear that with the fewer number of epochs, the same accuracy was achieved using CD.

**Fig. 7:**
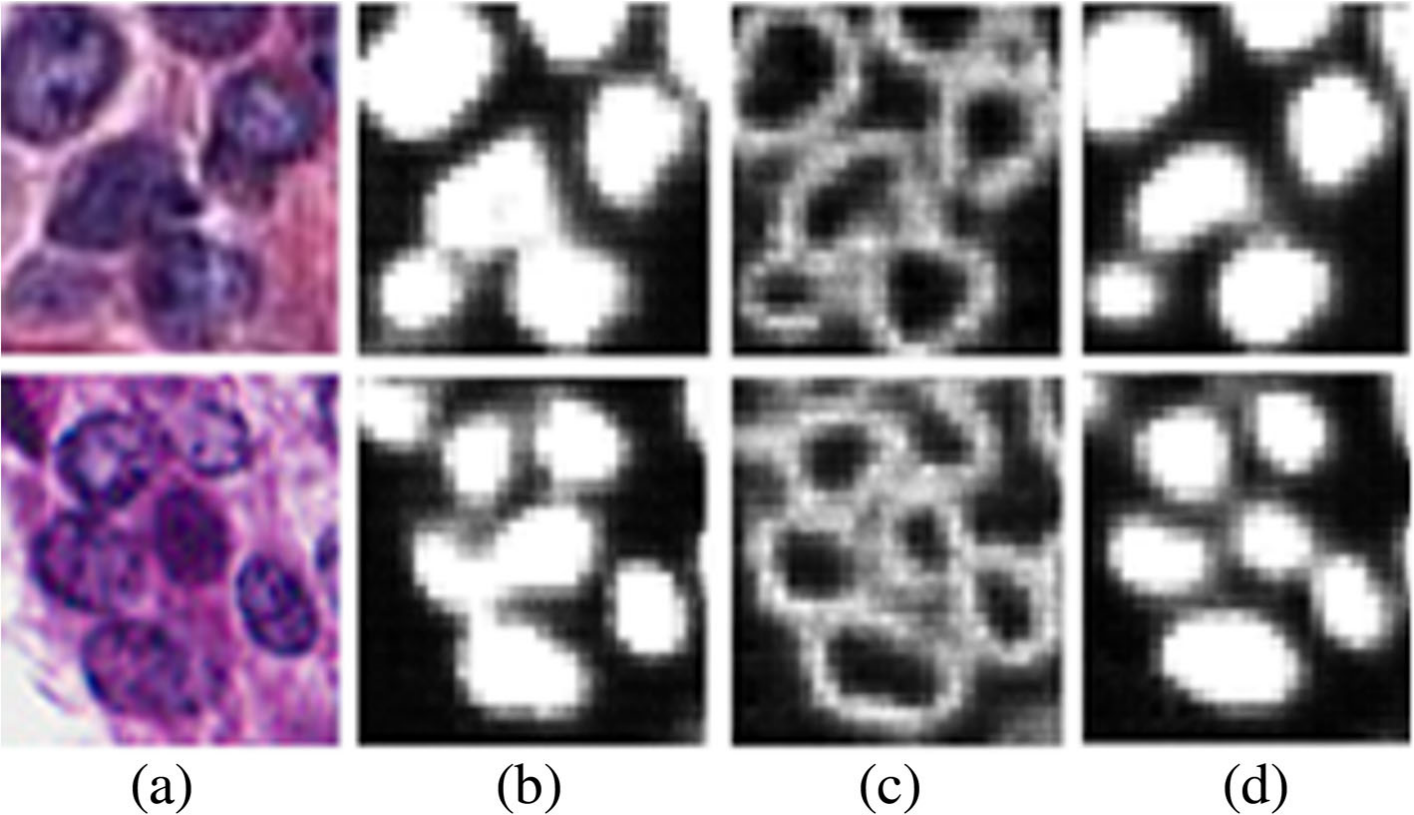
Integration of region-based and edge-based segmentation helps to separate touching nuclei. (**a**) shows two examples of touching and overlapping nuclei, (**b**) and (**c**) are the output probability map of the region-based and edge-based segmentation models, and (**d**) indicates the output probability map of the fusion model

*Fusion of region- and boundary-based ENets improves separation of overlapping nuclei:* The simulation results of Table 1 indicate that the three-layer network and ENet have the same performance profile for delineating nuclear regions. The next step is to improve separation of touching nuclei, and the best performing networks were selected to be trained with multiple representations of region- and boundary-based training followed by a fusion network. The results are shown in Table 2. The intent is to learn perceptual boundaries that aid in separation of touching nuclei. Interestingly, the ENet performed better than the three-layers of encoder-decoder architecture, which suggest that perceptual boundaries are higher order information and can only be learned with much deeper networks. To evaluate our approach, we randomly selected 98 touching nuclei from 16 independent images to conclude that 62 touching nuclei (e.g., 63.2% improvement) are correctly separated by fused ENet. The remaining touching nuclei can be delineated with the marker-based watershed. Qualitative representations of the fused ENet for delineating touching nuclei are visualized, for two test images, in Fig. 7.

*High speed segmentation is enabled:* The proposed model is time efficient and capable of performing instant-based segmentation. The efficiency is the result of (i) the model design and architecture, and (ii) using a GPU card for computations. The encoder-decoder architecture of the model allows for segmentation of the input image in one forward pass. In addition, small convolution operations (e.g., 1-by-1, 3-by-3, 5-by-1, 1-by-5) reduce the computational cost. The fusion network has been implemented on a server with a single GPU card. The processing time for an image of size 1k-by-1k is approximately 60 ms.

*Integration of nuclear detection does not improve segmentation results:* We evaluate the performance of the fusion model by applying the nuclear detection results with the region- and boundary-based information. The nuclei detection has been performed by using the method proposed in [22]. Our analysis indicated that the segmentation accuracy is not improved by adding the detection results.

## Conclusion

There are two intrinsic barriers to nuclear segmentation in H&E stained images. These are (i) the diversity of phenotypes and (ii) overlapping nuclei that form perceptual boundaries. We have demonstrated that some of these two issues can be largely resolved by the fusion of encoder-decoder architectures. The overall approach consists of color decomposition, training models for the region- and boundary-based representations, and a post-processing step. Color decomposition generates a single relevant gray scale image corresponding to the nuclear dye; hence, reducing the required number of training samples. This is significant since annotation is laborious and expensive. The encoder-decoder architecture enables region-based segmentation of complex nuclear phenotypes, which also includes the vesicular phenotype. Furthermore, by fusion of contour-based information, separation of adjacent nuclei is partially enabled. Some of the outstanding issues are in the choice of model (e.g., UNet, ENet) and the required number of layers for capturing the phenotypic diversities. We showed that by using the ENet for region-based segmentation, the learning rate plateaus as a function of increased number of layers, and the symmetric three layers network has the same performance of the original 17-layer asymmetric ENet. However, a fusion of region- and boundary-based models, with the original ENet, produces better results. In addition, (a) fusion of ENet performs better than the fusion of UNet, and (b) fusion has a better performance than multitask learning. The latter result must be due to the fact that sharing learned weights hinders the performance profile. The main insight is that there is a considerable amount of application-specific and data-specific variations that has a direct impact on the choice of model. Hence, a significant amount of simulation is needed for model selection. One of the main limitations of our approach is that the segmentation of touching nuclei is not fully resolved with the fusion of boundary- and region-based models. As a result, we still have to use the watershed method, which suffers from a number of limitations. Although we integrated and tested the detection step, from our previous work [22], segmentation was not improved. We suggest that perceptual boundaries are much higher level processes, which cannot be captured with the current network architectures and is the subject of our continued research. Finally, segmentation is fast and of the order of 20ms for an image size of 360-by-480 pixels running on a server with one GPU card. As a result, whole slide images can be processed efficiently and rapidly.

## Additional file

Additional file 1 Supplementary materials. (ZIP 120,330 kb)

## Abbreviations

CD: Color decomposition
CNN: Convolutional neural network
GMM: Gaussian mixture model
H&E: Hematoxylin and eosin
IOU: Intersection over union
LoG: Laplacian of gaussian
MRG: Multi reference graph cuts
SVM: Support vector machine
TCGA: The cancer genome atlas
WSI: Whole slide image
