# Supplementary material for: Fusion of encoder-decoder deep networks improves delineation of multiple nuclear phenotypes
Source: BMC Bioinformatics. 2018 Aug 7;19:294. doi: 10.1186/s12859-018-2285-0 (PMC6081825; doi:10.1186/s12859-018-2285-0)

Required libraries: Torch, image, cudnn, cutorch, cunn, nn

Region-based model:

File: model-seg.net

Input: RGB image

Code file: RegionEvaluation.lua

Edge-based model:

File: model-edge.net

Input: RGB image

Code file: EdgeEvaluation.lua

Fusion model:

File: model-fusion.net

Input: outputs of region-based and edge-based model

Code file: FusionEvaluation.lua

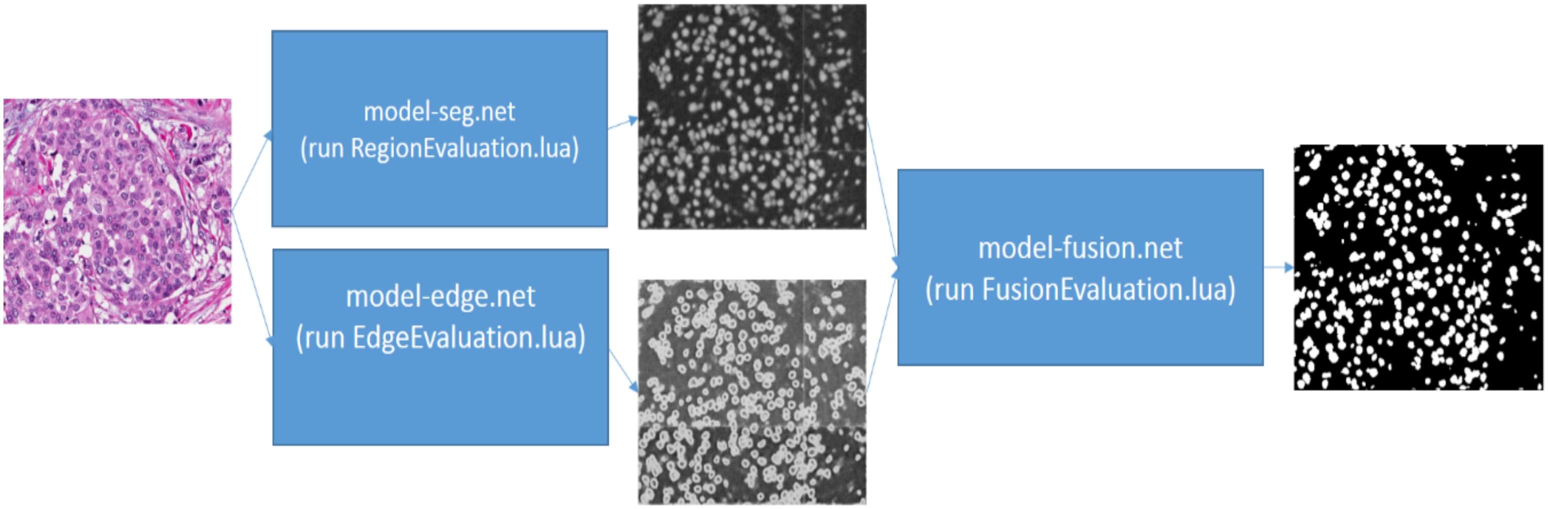

Supplement: Supplementary file 1 — Supplementary materials. (ZIP 120,330 kb) [file 12859_2018_2285_MOESM1_ESM.zip › 12859_2018_2285_MOESM1_ESM/Segmentation-master.zip!/Segmentation-master/quick guide.pdf]
